# Methodological challenges for conducting case-control studies to investigate the association between onchocerciasis and epilepsy including nodding syndrome

**DOI:** 10.1093/braincomms/fcad338

**Published:** 2023-12-20

**Authors:** Robert Colebunders, Amber Hadermann, Joseph Nelson Siewe Fodjo

**Affiliations:** Global Health Institute, University of Antwerp, Antwerp 2610, Belgium; Global Health Institute, University of Antwerp, Antwerp 2610, Belgium; Global Health Institute, University of Antwerp, Antwerp 2610, Belgium

We thank Edridge *et al*.^1^ for documenting their comprehensive research on the aetiology of nodding syndrome. However, we would like to comment on their methods and discussion of results obtained to avoid any misconceptions about epilepsy among affected communities in onchocerciasis-endemic areas. The fact that the authors did not find an association between nodding syndrome and onchocerciasis could be due to several methodological issues.

Regarding the study design, case-control studies that recruit nodding syndrome cases several years after seizure onset cannot adequately identify risk factors for developing nodding syndrome. A cohort study design is preferred to establish temporality of the suspected cause. Two such cohort studies were performed in Cameroon that showed a time-dependent and dose-related association between the *Onchocerca volvulus* microfilaria (mf) load in young children and the development of epilepsy later in life.^2^ Eldridge *et al*. attempted to solve the limitation of their cross-sectional design by recruiting nodding syndrome cases with seizure onset in the last 1 year. However, with the median age of nodding syndrome cases being 15 years (interquartile range: 9.75–17), it appears this criterion was not strictly respected, as the median age of nodding syndrome onset in Mundri is 8 years (interquartile range: 5–12).^3^ Therefore, most recruited nodding syndrome cases had their first seizures several years ago, before community-directed treatment with ivermectin (CDTI) restarted in the area in 2017.

Between 2001 and 2002, three case-control studies conducted by World Health Organization experts in the Lui Hospital (recruiting participants from the same sites as Edridge *et al*.) documented a very strong association between onchocerciasis and nodding syndrome [odds ratio (OR) > 9 in all study sites, 29 in Amadi]^4^ (Table 1). However, these contrasting findings were not discussed by the authors.

**Table 1:** Comparison of the 2001–02 and 2018–19 nodding syndrome case-control studies conducted at Lui Hospital, Mundri County, South Sudan

	2001–02 studies^4^	2018–19 studies^1,5^
Number of participants	82 cases and 94 controls^a^	72 cases and 109 controls
Selection process	House-to-house surveys	House-to-house surveys
Type of epilepsy	Nodding seizures exclusively	Nodding seizures or repeated generalized convulsions
History of ivermectin intake	In 2001: cases (61.5%) and controls (36.8%), OR: 2.79 (CI: 0.64–11.75)	Last 5 years: cases (64.3%) and controls (42.8%), OR: 2.40 (CI: 1.33–4.43)^5^
Proportion of skin snip positivity by microscopy	Lui (2001)^b^: cases (89.7%) and controls (48.3%), OR: 9.3 (CI: 0.64–11.75); Amadi (2001)^b^: cases (96.6%) and controls (76.4%), OR: 29.0 (CI:3.5–237.7); Lui (2002)^b^: cases (92.3%) and controls (43.7%), OR: 15.4 (CI: 1.6–148.8)	Median (interquartile range) microfilarial count^c^: cases 0 (0–25) and controls 0 (0–15), OR: 1.11 (CI: 0.98–1.27)
Proportion of skin snip positivity by PCR	Not tested	Cases (39%) and controls (30%), OR: 1.69 (CI: 0.82–3.47)
OV16 seropositivity	Not tested	Cases (62%) and controls (43%), OR: 1.94 (CI: 0.93–4.05)
*M. perstans* positivity by microscopy	Lui: cases (41.0%) and controls (96.6%); Amadi: cases (66.6%) and controls (50.0%), OR 3.22, *P* = 0.005	Median count: cases 0 (0–5) and controls 0 (0–3)
*M. perstans* positivity by PCR	Not tested	Cases (27%) and controls (5.7%), OR: 12.17 (CI: 2.76–53.77)

^a^Total number of cases enrolled during three case-control studies in Lui and Amadi (November 2001) and Lui (January 2002). ^b^Skin snips read after 24 hours incubation (World Health Organization guideline). ^c^Skin snips read after 1 h of incubation.

The main difference between the 2001 and 2002 nodding syndrome studies in Mundri and the recent 2018–19 study is that onchocerciasis was more prevalent in 2001–02 both among cases (>80%) and controls (reaching 76%). This can be explained by the fact that before 2001, there had been little ivermectin mass drug administration in Mundri and CDTI was only initiated in 2004. After several interruptions, CDTI was reintroduced in August 2017. Edridge *et al*.^1^ recruited participants from February 2018 to November 2019, a period during which two CDTI rounds were conducted (November 2018 and October 2019).

The two CDTI rounds in Mundri reduced onchocerciasis transmission without eliminating it. Single-dose ivermectin leads to decline in skin mf density by approximately 99% during the first 2 months. Therefore, to avoid the influence of ivermectin, a case-control study should ideally be conducted in an ivermectin-naïve population (Table 2), such as Ituri in the Democratic Republic of Congo.^6^ In such a setting, *O. volvulus* antibody and skin snip results strongly correlated with epilepsy.^6^ Although the authors refuted ivermectin use as a confounder, it should be considered as an effect modifier in nodding syndrome research, since it biases exposure to mf among participants prior to seizure onset.

**Table 2:** Recommendations to investigate the cause of epilepsy (including nodding syndrome) in onchocerciasis-endemic areas using case-control studies

Recommendation	Comment
Conduct study in places with high ongoing *O. volvulus* transmission	An ivermectin-naïve population is ideal, but it is unethical to not start CDTI in such places.
Choose age, sex and village-matched controls	Avoid family or close neighbour controls, as they may have a similar exposure to infected blackflies.
Enrol recent onset epilepsy cases	By enrolling cases many years after epilepsy onset, consequences of epilepsy rather than risk factors might be investigated.
Use a sensitive onchocerciasis antibody test	The OV16 ELISA test has low sensitivity and is affected by ivermectin use.
Determine mf load in skin snips after 24 h of incubation	Determining the mf load earlier leads to underestimation since mf have less time to emerge from the skin snips.
Obtain information about the last ivermectin intake and temporality of ivermectin use (before or after seizure onset)	People with epilepsy often take more ivermectin than controls.
Control for recent ivermectin use in analysis	Ivermectin eliminates 99% of mf during the first 2 months after ivermectin intake.
Obtain information about onchocerciasis clinical manifestations	In case ivermectin was used, a higher prevalence of nodules and sequalae of onchocerciasis skin lesion may still be observed.
Use a sufficiently large sample size	The sample size needs to be larger in areas with lower *O. volvulus* transmission, if ivermectin has been used, and/or if the *O. volvulus* laboratory test has low sensitivity.
Conduct case-control studies in different settings to identify common risk factors	Many risk factors were reported in the past because a very large number of risk factors were investigated, with some associations being spurious or lacking scientific logic. With the exception of *O. volvulus* infection and living/working close to (blackfly-infested) rivers, none of these risk factors were observed in all areas where nodding syndrome occurs.

The authors suggest that their results were not influenced by CDTI in the study area because an equal number of families of cases and controls were reported to have taken ivermectin. However, the individual ivermectin use of each family member was not recorded; neither was the temporality of ivermectin intake and nodding syndrome onset. However, ivermectin intake remains important for several reasons: (i) it plays a major preventive role as it depletes the *O. volvulus* parasitic load, thereby averting onchocerciasis-associated epilepsy (OAE) including nodding syndrome; (ii) after nodding syndrome onset, cases tend to take ivermectin more as they believe ivermectin may decrease seizure frequency^5^; (iii) ivermectin decreases onchocerciasis-induced troublesome itching, thereby preventing associated stress and sleep deprivation that could trigger seizures in persons with epilepsy.

The authors also claim no significant difference was observed concerning the seroprevalence of *O. volvulus* antibodies between cases and controls. Their data show a trend of higher OV16 seropositivity rates among nodding syndrome cases; however, only 63 (87.5%) of the 72 cases and 104 (95%) of the 109 controls were tested. Had all participants been tested at the same seropositivity rate (cases 62%; controls 43%), a significantly higher proportion of nodding syndrome cases would have been OV16 seropositive compared with controls (*P* = 0.01). In a much larger case-control study in Northern Uganda consisting of 154 nodding syndrome cases and 154 healthy community controls, cases had higher odds to present *O. volvulus* antibodies [OR 8.83, 95% confidence interval (CI): 4.48–17.86].^7^ In places with high onchocerciasis transmission, *O. volvulus* antibody testing and skin snip positivity may be of limited value to demonstrate an association between onchocerciasis and epilepsy; in such places, *O. volvulus* infection could be almost ubiquitous, and therefore, an association may only be demonstrated by a difference in mf load.^8^

Edridge *et al.* give as counter-argument for the *O. volvulus* nodding syndrome hypothesis that nodding syndrome has not been reported in many onchocerciasis-endemic regions. Indeed, nodding syndrome is only observed in high *O. volvulus* transmission zones that facilitate infections with sufficiently high mf loads that would trigger OAE including nodding syndrome in children. As a result of the large-scale implementation of CDTI, places with such characteristics are declining. Therefore, today nodding syndrome is mostly restricted to regions where onchocerciasis elimination programmes are either not operational or working sub-optimally, such as areas confronted with insecurity and war. In that regard, we strongly disagree with the authors’ statement that ‘premature public interventions were implemented despite that the proof of causality was lacking’. Nodding syndrome is a disease associated with severe disability, important psycho-social consequences and high mortality that needs to be prevented. Stronger onchocerciasis elimination interventions (e.g. switching from annual to bi-annual CDTI, with or without blackfly control) have contributed to significantly reduce epilepsy incidence in Northern and Western Uganda, Maridi (South Sudan) and Mahenge (Tanzania).^9^

Concerning *Mansonella perstans*, the 2018–19 findings were similar to those of a 2001–02 study.^4^ This was to be expected as there have been no interventions to control or treat *M. perstans*. *M. perstans* may be a co-factor in causing nodding syndrome in South Sudan but is unlikely to be the main or only causal factor as this parasite is not endemic in Mahenge, Tanzania, where nodding syndrome was first described.^10^ Moreover, in a cohort study in Cameroon, *M perstans* was not associated with the development of epilepsy in contrast with onchocerciasis.^2^ The reason for the concomitant presence of *O. volvulus* and *M. perstans* may be explained by filarial parasite tolerance induced in children following *in utero* exposure to onchocerciasis.^10^

Besides investigating the filarial risk factors, the authors also suggested that other factors such as higher vitamin A and E levels and fewer viral exposure could play a causative role in nodding syndrome. However, unlike *O. volvulus*, none of these factors meet any of the Bradford Hill criteria of causality. The most likely explanation for the high vitamin A and E levels is the administration of multi-vitamins to children with nodding syndrome, of whom 26% were malnourished (wasting).

An important limitation of the study is that little clinical information was collected. For example, cases and controls could have been examined for the presence of onchocerciasis nodules, itching, skin and eye lesions (Table 2). Moreover, no questionnaire about potential nodding syndrome risk factors was systematically administered to the study participants. Therefore, we lack information about the intake of ivermectin, other anti-parasitic drugs and vitamins; furthermore, no nutritional assessment was done.

We disagree with the authors to implement nodding syndrome preventive interventions only within the context of clinical trials. All children in at-risk communities should receive ivermectin at least annually. One example of an acceptable trial in nodding syndrome settings would be to compare annual versus 6-monthly intake of ivermectin. Such trial is currently being planned among children in areas of South Sudan, where bi-annual CDTI was hitherto impossible. However, such trials would require very large sample sizes and long follow-up periods to demonstrate a significant effect on epilepsy/nodding syndrome incidence. More importantly, we need to identify the pathogenesis of OAE. This will require more knowledge about the biology of *O. volvulus* and its interactions with the human host. Therefore, a series of omic studies have recently been initiated that include proteomics and viral metagenomics of *O. volvulus*.^9^

During the sixth meeting of the World Health Organization Onchocerciasis Technical Advisory Subgroup meeting in December 2022, it was stated that there was convincing evidence for the association between onchocerciasis and epilepsy (https://www.who.int/publications/i/item/9789240071469). Questioning this association and not recognizing OAE as an important preventable public health problem are very harmful. Indeed, risk communities should receive evidence-based information about OAE and should know there is a way to prevent their children from developing epilepsy/nodding syndrome. To call nodding syndrome, ‘a mysterious disease’ means that local populations will continue to associate epilepsy with bad spiritual forces, resulting in accrued epilepsy-related stigma and late initiation of anti-seizure medication because families will first seek care with traditional healers.

In the title of their paper, the authors imply that they investigated persons with nodding syndrome. However, as persons with epilepsy without head nodding seizures were also included, they mainly investigated persons with OAE. Recognizing OAE as part of the onchocerciasis disease burden will mobilize extra resources for onchocerciasis disease elimination and treatment and support for OAE-affected persons and families.

## Data Availability

Data sharing is not applicable to this article as no new data were created or analysed.
